# Resveratrol Enhances Cardiomyocyte Differentiation of Human Induced Pluripotent Stem Cells through Inhibiting Canonical WNT Signal Pathway and Enhancing Serum Response Factor-miR-1 Axis

**DOI:** 10.1155/2016/2524092

**Published:** 2015-12-21

**Authors:** Hui Liu, Shaoli Zhang, Lihua Zhao, Yan Zhang, Qiuping Li, Xiaoyan Chai, Yongchun Zhang

**Affiliations:** Department of Internal Medicine-Cardiovascular, First Affiliated Hospital of Xinxiang Medical University, Weihui, Henan 453100, China

## Abstract

Resveratrol (trans-3,5,4′-trihydroxystilbene) (RSV) is a natural polyphenol with protective effects over cardiac tissues and can affect cell survival and differentiation in cardiac stem cells transplantation. However, whether this agent can affect cardiomyocytes (CMs) differentiation of induced pluripotent stem cells (iPSCs) is not yet clear. This study explored whether RSV can affect CMs differentiation of human iPSCs. Under embryoid bodies (EBs) condition, the effect of RSV on the change of pluripotent markers, endoderm markers, mesoderm markers, and ectoderm markers was measured using qRT-PCR. Under CM differentiation culture, the effect of RSV on CM specific markers was also measured. The regulative role of RSV over canonical Wnt signal pathway and serum response factor- (SRF-) miR-1 axis and the functions of these two axes were further studied. Results showed that RSV had no effect on the self-renewal of human iPSCs but could promote mesoderm differentiation. Under CM differentiation culture, RSV could promote CM differentiation of human iPSCs through suppressing canonical Wnt signal pathway and enhancing SRF-miR-1 axis.

## 1. Introduction

Due to the unlimited proliferation potential and differentiation capacity, induced pluripotent stem cells (iPSCs) have drawn wide attention as a promising source of cellular therapy. Some recent studies suggest that transplantation of cardiomyocytes (CMs) differentiated from human iPSCs can be a promising treatment of acute myocardial infarction [1, 2]. In fact, iPSCs must be predifferentiated into cardiovascular precursors or CMs in vitro to avoid teratoma formation in vivo [3]. Targeted production of CMs is therefore quite necessary [4]. Some recent studies suggest that small molecular compounds may play an important role in the process of target differentiation of iPSCs [5, 6].

Resveratrol (trans-3,5,4′-trihydroxystilbene) (RSV) is a natural polyphenol found in various plants [7]. A series of researches demonstrated that RSV possesses various health-benefiting effects, such as anti-inflammatory, antioxidative, antiaging, and anticancer effects [8, 9]. In rats with myocardial infarction and received cardiac stem cells transplantation, RSV improves therapeutic effects by enhancing cell survival and differentiation [10, 11]. Previous studies showed that RSV can promote osteogenic differentiation of murine induced pluripotent stem cells [12]. Although this agent shows protective effects over cardiac tissues, whether it affects differentiation of cardiovascular progenitor cells is not clear. Some recent studies showed that RSV can suppress Wnt/beta-catenin signaling pathway in several diseases [13–15]. In fact, the canonical Wnt signaling plays a vital role in CM differentiation of human iPSCs [5, 16]. Besides, RSV functions as prodifferentiating agent promoting differentiation of mouse skeletal muscle-derived C2C12 myoblasts partly through enhancing serum response factor (SRF) transcription [17]. MiR-1, a SRF dependent miRNA, is highly expressed in cardiomyocytes and their precursors and regulates cardiomyogenesis [18–20]. In addition, SRF-MiR-1 is proved to be an important molecular axis modulating mesodermal and cardiac fate of pluripotent stem cells [18, 21]. Therefore, it is quite possible that RSV, as a canonical Wnt signaling suppressor and upstream regulator of SRF, might affect CM differentiation of human iPSCs.

In this study, we explored the effect of RSV in CM differentiation of human iPSCs and firstly reported that RSV could enhance the differentiation through inhibiting canonical Wnt signal pathway and enhancing SRF-miR-1 axis.

## 2. Method

### 2.1. Cell Culture

Human iPSC line DYR0100 was obtained from the Type Culture Collection of the Chinese Academy of Sciences, Shanghai, China. The cells were maintained on mitomycin-C treated mouse embryonic fibroblasts (MEF) and cultured in DMEM/F12 supplemented with 20% knock-out serum replacement (Invitrogen) with 10 ng/mL human basic fibroblast growth factor at 37°C in a 5% CO_2_ incubator.

To explore the effect of RSV on cell proliferation, the iPSCs were maintained on BD Matrigel, cultured in mTeSR1 (#05850, Canada Stemcell), and treated with 0, 10, 50, or 100 *μ*M RSV, respectively, for 5 days. Morphologic shape of iPSCs was checked under a light microscope at 10x magnification and cell proliferation rate was measured by Cell Counting Kit-8 (CCK-8) (Dojindo, Japan) according to the manufacturer's instruction. 50 *μ*M RSV was used for the remaining functional studies.

### 2.2. Cell Transfection and Reagents

Human SRF shRNA lentiviral particles and the control shRNA lentiviral particles were purchased from OriGene (TL320530). To knock down endogenous SRF, iPSCs were treated with SRF shRNA lentiviral particles with the presence of 5 *μ*g/mL Polybrene (sc-134220, Santa Cruz) according to the recommended manual. To knock down endogenous miR-1, iPSCs were transfected with 200 nM anti-miR-1 (sequence: ACAUACUUCUUUAUAUGCCCAU, Ambion, Life Technology) using Lipofectamine 2000 reagent (Invitrogen). The ratios of beating EBs formed by iPSCs with miR-1 or SRF knockdown alone or with knockdown of miR-1 and SRF in combination on day 24 were calculated.

### 2.3. Cardiovascular Differentiation Strategy of iPSCs

To form hanging drop embryoid bodies (EBs), dissociated iPSC clumps were plated in 96-well U bottom plate (174925, Thermo Scientific). EBs were treated with the following conditions at indicated time period: days 0–5, BMP4 (10 ng/mL); days 2–5, RSV (0, 5, 25, or 50 *μ*M), activin A (3 ng/mL), and bFGF (5 ng/mL). The medium was renewed every two days. The ratios of beating EBs on days 8, 12, 16, 20, and 24 were calculated. To explore the influence of canonical Wnt signal on CM differentiation of iPSCs, 0, 25, 50, or 100 *μ*M Wnt3A (R&D Systems) or 0, 25, 50, or 100 *μ*M DKK1 (R&D Systems) was added to the culture from days 2 to 5. Ratios of beating EBs on day 24 were calculated.

### 2.4. qRT-PCR Analysis

Total RNAs from cell samples were extracted using a miRNeasy Mini extraction kit (Qiagen) and the cDNA was reversely transcribed using a First-Strand Synthesis kit (Invitrogen). qRT-PCR analyses of pluripotent markers (Oct4, Sox2, NANOG, and KLF4), endoderm markers (FOXA2, AFP, and SOX17), mesoderm markers (MESP1, Brachyury, and Mixl1), ectoderm markers (SOX1, PAX6, and Notch1), CM specific markers during cardiovascular differentiation (NKX2.5, GATA4, cTnT, *α*-MHC, and *β*-MHC), and the transcription factor-serum response factor (SRF) were performed using gene specific primers (see supplementary Table 1 in Supplementary Material available online at http://dx.doi.org/10.1155/2016/2524092) and Power SYBR Green PCR Master Mix in an ABI Prism 7300 (Applied Biosystems). Results were normalized to GAPDH transcripts and analyzed using 2^−ΔΔ*Ct*^ method.

To quantify miR-1 expression in iPSCs treated with RSV, total RNAs were extracted using the mirVana PARIS Kit (Ambion, USA). Then, the miRNA specific cDNA was reversely transcribed using the TaqMan MicroRNA Reverse Transcription Kit. To quantify the expression of miR-1, TaqMan MicroRNA Assay Kit (Applied Biosystems) was used. U6 snRNA served as the internal control. qRT-PCR was performed in an ABI PRISM 7300 (Applied Biosystems).

### 2.5. Immunofluorescent Staining

Cells were fixed with 4% (vol/vol) paraformaldehyde for 15 min at room temperature and then stained with anti-cTnT (1 : 500, ab8295, Abcam) and secondary antibodies (1 : 500, ab150113, goat anti-mouse IgG H&L (Alexa Fluor 488)) in PBS plus 0.4% (vol/vol) Triton X-100 and 5% (wt/vol) nonfat dry milk (Bio-Rad). Nuclei were stained with Gold Antifade Reagent with DAPI (Invitrogen). The signals were visualized using a Ti-S inverted phase/fluorescent microscope with SPOT cooled 2.0-megapixel digital camera system (Nikon).

### 2.6. Flow Cytometry Analysis

Cells were harvested and rinsed three times with cold PBS and then fixed with 0.5% paraformaldehyde (PFA) for 20 min and permeabilized with 0.1% Triton X-100. Then, the cells were stained with anti-cTnT (1 : 500, ab10214, Abcam) followed by an Alexa Fluor 594 conjugated secondary antibody. cTnT positive cells were measured using a FACSCalibur flow cytometer (BD Bioscience, CA, USA).

### 2.7. Luciferase Reporter Assay

iPSCs were cotransfected with TOP Flash or FOP-Flash constructs (Addgene) and a Renilla luciferase plasmid (Promega). Then, the iPSCs were seeded for the formation of EBs. The EBs were treated with 0, 5, 25, or 50 nM RSV. On day 4, cells were harvested and lysed. The relative luciferase activity of the lysate was measured with the Dual-Luciferase reporter assay system (Promega).

### 2.8. Western Blot Analysis

Cells were lysed using a lysis buffer (Beyotime, China) and the lysates were separated on 10% SDS-PAGE. The proteins on the gel were then transferred onto a PVDF membrane. After blocking with 5% nonfat dry milk, the membranes were incubated with primary antibodies (1 : 1000, anti-cTnT, ab8295; anti-NKX2.5, ab106923; anti-GATA4, ab86371; anti-*α*-MHC, ab50967; anti-*β*-MHC; ab172967, Abcam; anti-SRF, MAB4369; anti-GAPDH, ABS16, Merck Millipore) overnight at 4°C. Membranes were washed and incubated with corresponding HRP-labeled secondary antibodies (anti-mouse IgG, 1: 10000, 71045; anti-rabbit IgG, 1 : 10000, AP188P, Merck Millipore). The band signals were visualized using the ECL Western Blotting substrate (Pierce) and the signal intensity was quantified using ImageQuant 5.2 (GE Healthcare, Piscataway, NJ). To clearly demonstrate the difference, the relative gray-scale value of target protein and internal control of control group was set as 1.

### 2.9. Intracellular Ca^2+^ Measurements

To record calcium transients, the cardiomyocytes were loaded with 5 *μ*M Fluo-4 AM Ca^2+^ indicator plus PowerLoad (Invitrogen Corp.) according to the manufacturer's instruction. Spontaneous Ca^2+^ transients were recorded at 30°C via a standard optical filter set (Chroma, Bellows Falls, VT, USA) using a PMT400 system (IonOptix, Milton, MA, USA). In CM clusters, fluorescence was recorded from the entire cluster in addition to a cell-free border via adjusting a cell-framing adaptor. Between sampling periods, excitation light was blocked by a shutter (CS35; Vincent Associates, Rochester, NY, USA) and background fluorescence was recorded after removing the cell(s) from the field of view at the end. Where appropriate, 1 *μ*M Iso (isoproterenol) (Sigma, St. Louis, MO, USA) or 25 *μ*M CCH (carbamylcholine chloride) (Sigma, St. Louis, MO, USA) was applied locally to the cell or cluster of interest using a perfusion pencil (AutoMate Scientific, Berkeley, CA, USA).

### 2.10. Statistical Analysis

Data analysis was performed using SPSS 17.0. Data were presented as mean ± standard deviation (SD). Group difference was evaluated using Student's *t*-test. *P* < 0.05 was regarded as statistically significant. *∗* and *∗∗* denote significance at 0.05 and 0.01 level, respectively.

## 3. Results

### 3.1. RSV Has No Effect on the Self-Renewal of Human iPSCs but Can Promote Mesoderm Differentiation

**Figure 1:**
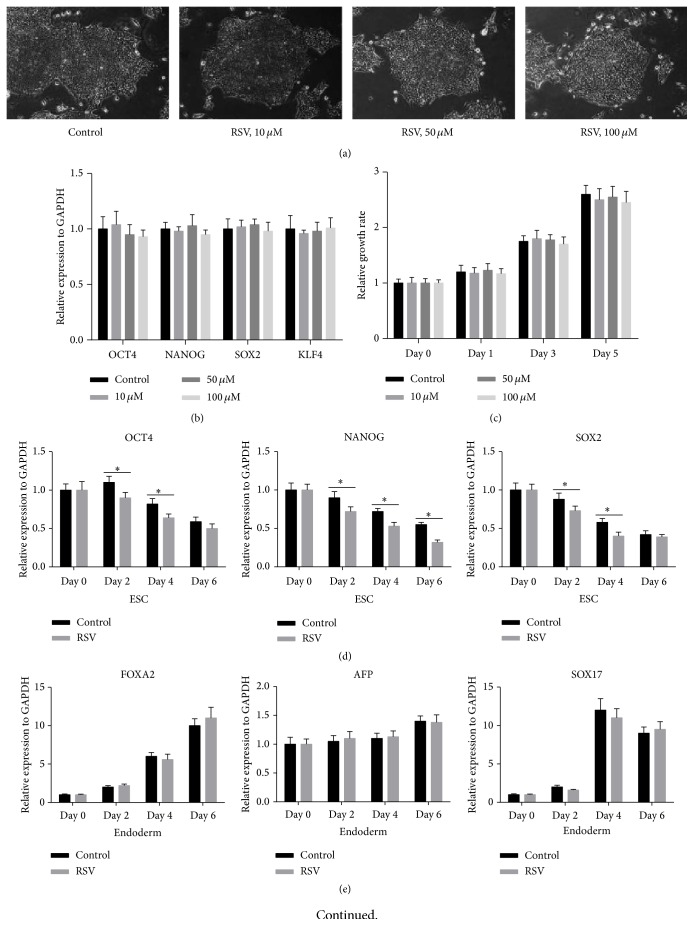
RSV has no effect on the self-renewal of human iPSCs but can promote mesoderm differentiation. (a) Representative images of the human iPSCs morphology 5 days after different concentrations of resveratrol treatment (10x). (b) Quantitative RT-PCR (qRT-PCR) analysis of pluripotent markers Oct4, Sox2, NANOG, and KLF4 in human iPSCs 5 days after RSV administration. (c) Cell proliferation assay was performed using CCK-8. (d–g) Under embryoid body (EB) condition, dynamic gene expressing profile of pluripotency (d), endoderm (e), mesoderm (f), and ectoderm markers (g) were detected by qRT-PCR analysis. Data represent the mean ± s.d. of three biological replicates. ^*∗*^ *P* < 0.05.

To study the effect of RSV on human iPSCs under maintenance condition, the cells were firstly treated with 0, 10, 50, or 100 *μ*M of RSV for 5 days. The morphologies of the colonies were similar among different RSV concentration groups (Figure 1(a)). Then, we quantified the expression of pluripotent markers Oct4, Sox2, NANOG, and KLF4 in human iPSCs 5 days after RSV administration. qRT-PCR results suggested that there were no significant differences among different RSV concentration groups (Figure 1(b)). In addition, we measured growth rate of the cells at days 0, 1, 3, and 5, but we found no significant difference (Figure 1(c)). These results indicate that RSV had no effect on the self-renewal of the cells under the maintenance condition. Under EB condition, we quantified the expression of endoderm markers (FOXA2, AFP, and SOX17), mesoderm markers (MESP1, Brachyury, and Mxl1), and ectoderm markers (SOX1, PAX6, and Notch1). qRT-PCR results showed that 50 nM RSV treatments had no effect on endoderm and ectoderm markers (Figures 1(e) and 1(g)), but they decreased the expression of ESC markers OCT4, NANOG, and SOX2 (Figure 1(d)) and promoted the expression of mesoderm markers, MESP1, Brachyury, and mixl1 significantly (Figure 1(f)). These results suggest that RSV can promote mesoderm differentiation of iPSCs under EB condition.

### 3.2. RSV Enhances CM Differentiation of Human iPSCs

**Figure 2:**
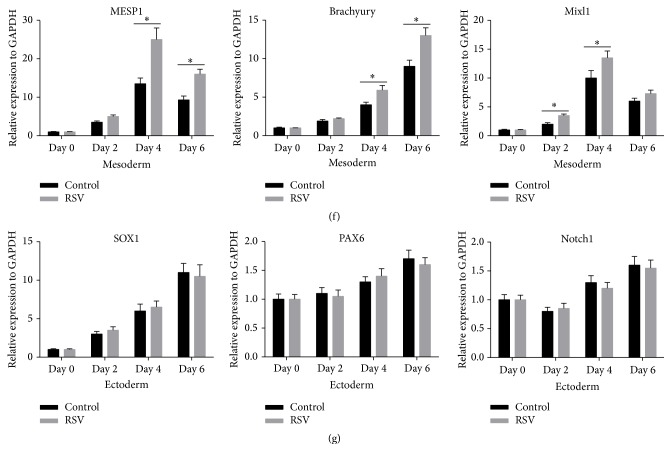
RSV enhances CM differentiation of human iPSCs. (a) A protocol outlined describing the cardiovascular differentiation strategy. (b) Ratios of beating EBs in control and RSV group from day 8 to day 24 of differentiation, respectively (*n* = 100). (c) Gene expression profile of pluripotency, cardiomyocyte specific markers during differentiation process by qRT-PCR analysis. (d) A representative immunofluorescent image of comparison of cTnT positive CMs from dissociated day 24 EBs between control and RSV groups (40x). (e) Flow cytometry was performed to compare the percentage of cTnT positive cells between control and RSV group at day 24. (f) cTnT protein level was further compared using Western Blot method at day 24. (g) Western Blot analysis was further performed to confirm the cardiomyocyte specific genes (as shown in (c)) at protein levels. Data represent the mean ± s.d. of three biological replicates. ^*∗*^ *P* < 0.05, ^*∗∗*^ *P* < 0.01.

Considering the role of RSV in promoting mesoderm differentiation of iPSCs, we then developed a protocol to further explore how RSV affects CM differentiation of the cells. The contracting cardiomyocytes generated by EB methods possess typical cardiac electrophysiological features. For example, typical Ca^2+^ transients, which underlie the contraction and relaxation of spontaneously beating cardiomyocytes, were detected (Supplementary Figure 1A). The ability to respond appropriately to hormones and transmitters of the autonomic nervous system is one critical feature of normal cardiomyocytes. The addition of Iso, a *β*-adrenergic agonist, significantly increased the frequency of cell contractions and spontaneous calcium transients, while administration of CCH, a muscarinic agonist, decreased the frequency of cell contractions and spontaneous calcium transients (Supplementary Figures 1B, C, and D). After washing out CCH, cells immediately resumed contracting at baseline frequency (Supplementary Figure 1D). The EBs form in suspension condition before day 5 and were then replated to adherence condition (Figure 2(a)). Based on this protocol, we measured the ratio of beating EBs in control and RSV group from day 8 to day 24 since differentiation. On days 12, 16, 20, and 24, the ratios of beating EBs in RSV groups were significantly higher than those of the control group (Figure 2(b)). During the CM differentiation process, we also measured the pluripotency markers, OCT4 and NANOG, and CM specific markers, NKX2.5, GATA4, cTnT, *α*-MHC, and *β*-MHC. qRT-PCR analysis demonstrated that RSV treatment significantly reduced OCT4 and NANOG expression at day 2 and day 8 (Figure 2(c)), but it substantially enhanced the expression of cardiac-specific genes NKX2.5, GATA4, cTnT, *α*-MHC, and *β*-MHC since day 8 (Figure 2(c)). Western Blot analysis also confirmed enhanced protein expression of these genes on day 24 (Figure 2(g)). In addition, the iPSCs cells treated with RSV also had stronger cTnT expression (Figures 2(d), 2(e), and 2(f)), suggesting enhanced direct differentiation into CMs. These results show that RSV can enhance CM differentiation of human iPSCs.

### 3.3. RSV Enhances CM Differentiation Partially through Inhibiting Canonical WNT Signal Pathway

**Figure 3:**
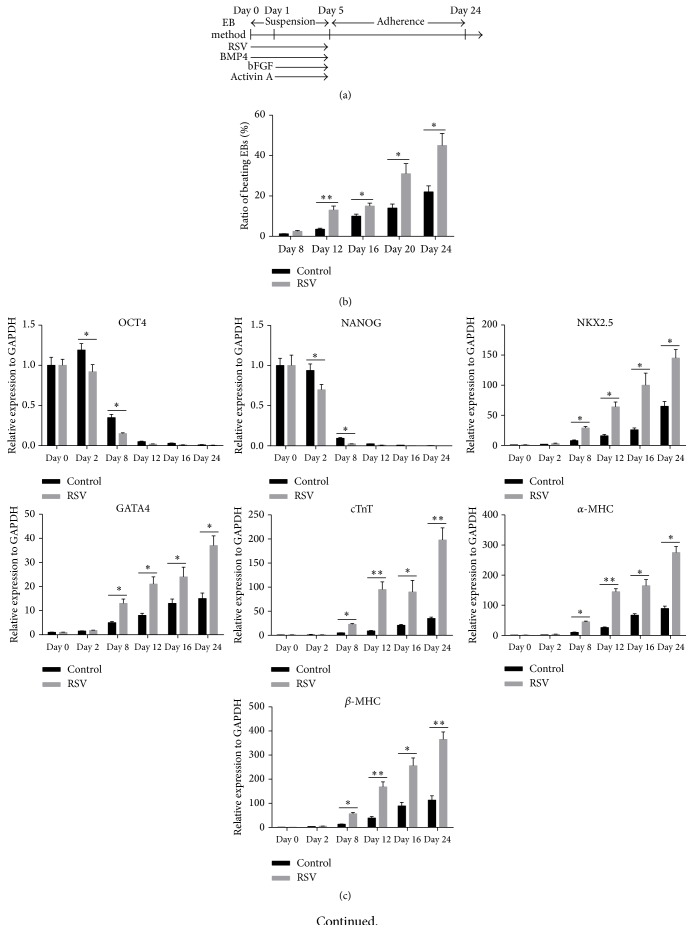
RSV enhances CM differentiation partially through inhibition of WNT signal pathway. (a) An outline describing the administrations of pathway modulators during CM differentiation. (b) TOP Flash assay of human iPSCs treated with different concentrations of RSV in suspension culture condition at day 4. (c) From day 0 to day 5, TOP Flash assays were performed, respectively, after 50 *μ*M RSV incubation. (d, e). The effects of different concentrations of DKK1, WNT inhibitor (d) and WNT3A, WNT activator (e) on the ratio of beating EBs were evaluated on day 24 (*n* = 100). Data represent the mean ± s.d. of three biological replicates. ^*∗*^ *P* < 0.05 compared with control; ^#^ *P* < 0.05 compared with RSV alone.

Since we verified the effect of RSV on enhancing CM differentiation of human iPSCs, we continued to explore the underlying mechanism. Since canonical Wnt signal has a well-recognized role in CM development, we designed a protocol to investigate its involvement in CM differentiation of human iPSCs (Figure 3(a)). Canonical WNT signaling is defined by an accumulation of *β*-catenin in the cytoplasm, while noncanonical WNT signaling does not. By performing TOP Flash assay which depends on activity of *β*-catenin, we observed that iPSCs treated with 25 *μ*M or 50 *μ*M RSV had significantly lower relative TOP/FOP luciferase activity, suggesting the inhibition of the canonical WNT signal pathway (Figure 3(b)). To further confirm the inhibiting effect, TOP Flash assay was also performed in iPSCs treated with 50 *μ*M RSV from day 0 to day 5. The results showed that since day 2 the cell lysate had significantly lower relative TOP/FOP luciferase activity than that of day 0 (Figure 3(c)). To verify the effect of canonical WNT signal pathway in CM differentiation, WNT3A, an activator of Wnt/*β*-catenin pathway, or DKK1, an antagonist, the pathway was added to the cell culture since day 2. Results showed that 50 and 100 *μ*M DKK1 significantly increased the ratio of beating EBs. Additionally, 50 and 100 *μ*M DKK1 also significantly enhanced RSV's effect in promoting beating EBs (Figure 3(d)). In contrast, 50 and 100 *μ*M WNT3A substantially decreased the ratio and also weakened the effect of RSV (Figure 3(e)). In combination, these results indicate that RSV can enhance CM differentiation partially through inhibiting canonical WNT signal pathway.

### 3.4. SRF-miR-1 Axis Is Also Involved In the CMs Differentiation Enhanced by RSV

**Figure 4:**
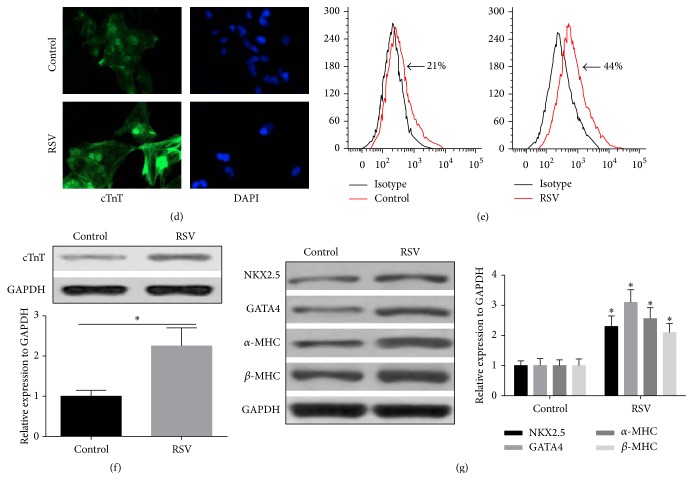
Serum response factor- (SRF-) miRNA-1 axis also involves the CMs differentiation enhanced by RSV. (a) Transcription levels of SRF in cultured EBs treated with RSV (50 *μ*M) were detected by qRT-PCR on day 4. (b, c) Western Blot was further carried out to determine the protein levels of RSV (50 *μ*M) treated EBs after 4 days. (d) miRNA-1 expression level was also detected using qRT-PCR 4 days after RSV (50 *μ*M) administration. (e, f) Knockdown of SRF using produced* Lentivirus* expressing shRNA targeting SRF was confirmed by Western Blot analysis. (g) SRF-miRNA-1 signal axis was confirmed in EBs treated with RSV (50 *μ*M) on day 4. (h) Inhibition of endogenous miRNA-1 in floating cultured EBs 4 days after transfection of* Lentivirus* within anti-miRNA-1. (i) The effects of modulation of SRF or/and miRNA-1 on the ratio of beating EBs were evaluated on day 24 (*n* = 100). Data represent the mean ± s.d. of three biological replicates. ^*∗*^ *P* < 0.05 compared with control; ^#^ *P* < 0.05 compared with RSV alone.

To explore whether SRF-miR-1 axis is involved in RSV enhanced CM differentiation, transcription and protein levels of SRF in EBs treated with 50 *μ*M RSV were detected on day 4. RSV remarkably promoted SRF expression at both mRNA and protein levels (Figures 4(a), 4(b), and 4(c)). qRT-PCR analysis also revealed that 50 *μ*M RSV administration enhanced miR-1 expression (Figure 4(d)). To further investigate the function of SRF and miR-1, iPSCs were transfected with si-SRF (Figures 4(e) and 4(f)) or anti-miR-1 (Figure 4(h)). Knockdown of endogenous SRF partly abrogated RSV's effect in enhancing miR-1 expression (Figure 4(g)). Blocking either SRF or miR-1 substantially weakened the effect of RSV in CMs differentiation, while blocking both showed stronger weakening effect. (Figure 4(i)). These results indicate that the SRF-miR-1 axis also involved the CMs differentiation enhanced by RSV.

## 4. Discussion

Currently, supplementation of soluble factors to culture medium and using mechanical stimulation were the main methods used to drive differentiation of pluripotent stem cells into mesodermal lineages [22, 23]. The effect of RSV on direct differentiation of iPSCs or human embryonic stem cells (hESCs) was reported in previous studies. For example, RSV can facilitate differentiation of iPSCs into osteocyte-like cells and decrease tumorigenicity of iPSCs [12]. Another recent study observed that RSV treatment improves the maturation process of pancreatic organogenesis of hESCs and helps to obtain functional insulin-secreting cell in vivo [24]. In the current study, we found that RSV can promote mesoderm differentiation of iPSCs. What is more, under the CM differentiation culture, RSV supplementation significantly enhanced the differentiation. Additionally, we found the EBs treated with RSV from day 2 to day 5 enhanced the expression of cardiomyocyte specific markers and also increased the proportion of beating EBs.

Due to the remarkable enhancing effect, we explored the underlying molecular mechanism. The canonical Wnt signaling plays a vital role in CM differentiation of human iPSCs [5, 16]. During early embryonic differentiation, Wnt signal is necessary for mesodermal specification. However, later on, CM differentiation is hampered by WNT signaling [25]. Therefore, early activation of Wnt and then Wnt inhibition support CM differentiation from iPSC lines [16, 26]. One recent study found that inhibition of the Wnt/*β*-catenin pathway by small molecule enhanced the differentiation of cardiac progenitors to ventricular cardiomyocyte lineage [27]. Another recent study reported that induction of WNT signaling via the GSK3 inhibitor for 2 days enables efficient differentiation of human monolayer pluripotent stem cells to endothelial progenitors [6]. Inhibition of Wnt between days 3 and 8 since differentiation results in optimal cardiac differentiation [26]. In combination, through modulating Wnt/*β*-catenin pathway, it is possible to induce direct differentiation of iPSCs into CMs.

The regulative effect of RSV over Wnt/*β*-catenin pathway has been reported in previous studies. For example, in human cervical cancer cells, colon cancer cells, RSV presented antiproliferative and apoptosis-inducing activities partly through suppressing Wnt/beta-catenin signaling [15, 28]. RSV can also inhibit breast cancer stem-like cells and it induces autophagy via repressing Wnt/beta-catenin signaling [14]. Therefore, RSV has general inhibiting effect on canonical Wnt pathway. In this study, we found that the canonical Wnt signal intensity was negatively correlated to RSV concentration, suggesting that there is a dose dependent suppressing effect. Besides, under the treatment of 50 *μ*M RSV, the Wnt signal from day 2 to day 5 was significantly weaker than that of day 1. Therefore, RSV is an agent with potent suppressing effect on canonical Wnt signal in iPSCs. Additionally, we also found that the use of WNT3A, an activator of canonical Wnt, significantly weakened the effect of RSV. But supplementation of DKK1, a canonical Wnt inhibitor, significantly enhanced the effect of RSV.

Previous studies also reported that miRNAs participate in modulating cardiac linage commitment in pluripotent stem cells, including both ESCs and iPSCs [18, 21, 29]. MiR-1 plays an important part in CM commitment from human cardiovascular progenitors via suppressing both WNT and FGF signaling pathways [30]. In that study, the authors demonstrated that 100 ng Wnt3A completely suppressed CM differentiation in miR-1 overexpressing iPSCs [30], suggesting that miR-1 mediates CM differentiation via antagonizing the suppressor role of Wnt3A. In fact, miR-1 is a SRF dependent microRNA [18–20]. Inhibiting SRF directly led to decreased miR-1 expression in the heart of mice [20]. RSV is a demonstrated agent promoting differentiation of mouse myoblasts partly through enhancing SRF transcription [17]. In the current study, we also found that RSV exerted strong regulation over SRF expression and subsequent miR-1 expression. RSV treatment could enhance SRF expression at both mRNA and protein levels and further increase miR-1 expression. Through regulating SRF-miR-1 axis, RSV increased the ratio beating EBs of human iPSCs.

This study also has a few limitations. Firstly, besides the canonical Wnt/*β*-catenin pathway, RSV may also regulate noncanonical Wnt pathway, such as Wnt5a [31]. Till now, at least 19 Wnt proteins were identified, forming a complex regulative network [32]. In this study, we only focused on the canonical Wnt/*β*-catenin pathway. But it is of great importance to further study the wide and detailed effect of RSV on both canonical and noncanonical Wnt signaling in CM differentiation of iPSCs. Secondly, one recent study found that miR-1 mediates CM differentiation via antagonizing the suppressor role of Wnt3A [30]. In combination with our data, it is possible that RSV's suppressive effect over canonical Wnt is partially through increasing miR-1 expression, which antagonizes Wnt3A. However, the canonical pathway involves other Wnt proteins, such as wnt1, wnt2, wnt3, wnt8, and wnt8a [32]. Since RSV has general inhibiting effect on canonical Wnt pathway, we could not exclude the possibility that, besides the linear RSV-SRF-miR-1-Wnt3A pathway, RSV may exert differentiation inducing effect through other Wnt proteins or even non-Wnt pathways. In fact, our preliminary work showed that SRF-miR-1 might exert function through non-Wnt pathways. Therefore, the role of RSV in CM differentiation might be more complex than we reported in this study.

## 5. Conclusion

In conclusion, RSV is an agent with potent suppressing effect on canonical Wnt signal and exerts strong regulation over SRF expression and subsequent miR-1 expression in iPSCs. RSV enhances CM differentiation of human iPSCs at least partially through these two pathways.

## Supplementary Material

Supplementary table 1: Primers used for qRT-PCR analysis.
Supplementary figure 1 Contracting cardiomyocytes generated by EB methods exhibit typical calcium movement and sensitive response to both cardiomyocyte agonist and antagonist.
(A) Spontaneously contracting cardiomyocytes differentiated were recorded for the characteristic calcium transients ([Ca2+]i), and the representative curve was showed. (B) The effects of 1mM isoproterenol (Iso) (E and F) and 25mM carbachol (CCH) on the beating rates of enabled cardiomyocytes . (C, D) Calcium transients were compared before and after treatment 1mM Iso (C) and 25mM CCH) (G). Data are mean ± s.d. ∗ P<0.05 compared with control.
