# Supplementary material for: Resveratrol Enhances Cardiomyocyte Differentiation of Human Induced Pluripotent Stem Cells through Inhibiting Canonical WNT Signal Pathway and Enhancing Serum Response Factor-miR-1 Axis
Source: Stem Cells Int. 2015 Dec 21;2016:2524092. doi: 10.1155/2016/2524092 (PMC4699094; doi:10.1155/2016/2524092)
Supplement: Supplementary file 1 — Supplementary table 1: Primers used for qRT-PCR analysis. Supplementary figure 1 Contracting cardiomyocytes generated by EB methods exhibit typical calcium movement and sensitive response to both cardiomyocyte agonist and antagonist. (A) Spontaneously contracting cardiomyocytes differentiated were recorded for the characteristic calcium transients ([Ca2+]i), and the representative curve was showed. (B) The effects of 1mM isoproterenol (Iso) (E and F) and 25mM carbachol (CCH) on the beating rates of enabled cardiomyocytes . (C, D) Calcium transients were compared before and after treatment 1mM Iso (C) and 25mM CCH) (G). Data are mean ± s.d. ∗ P<0.05 compared with control. [file 2524092.f1.pdf]

Supplementary figure 1 Contracting cardiomyocytes generated by EB methods exhibit typical calcium movement and sensitive response to both cardiomyocyte agonist and antagonist

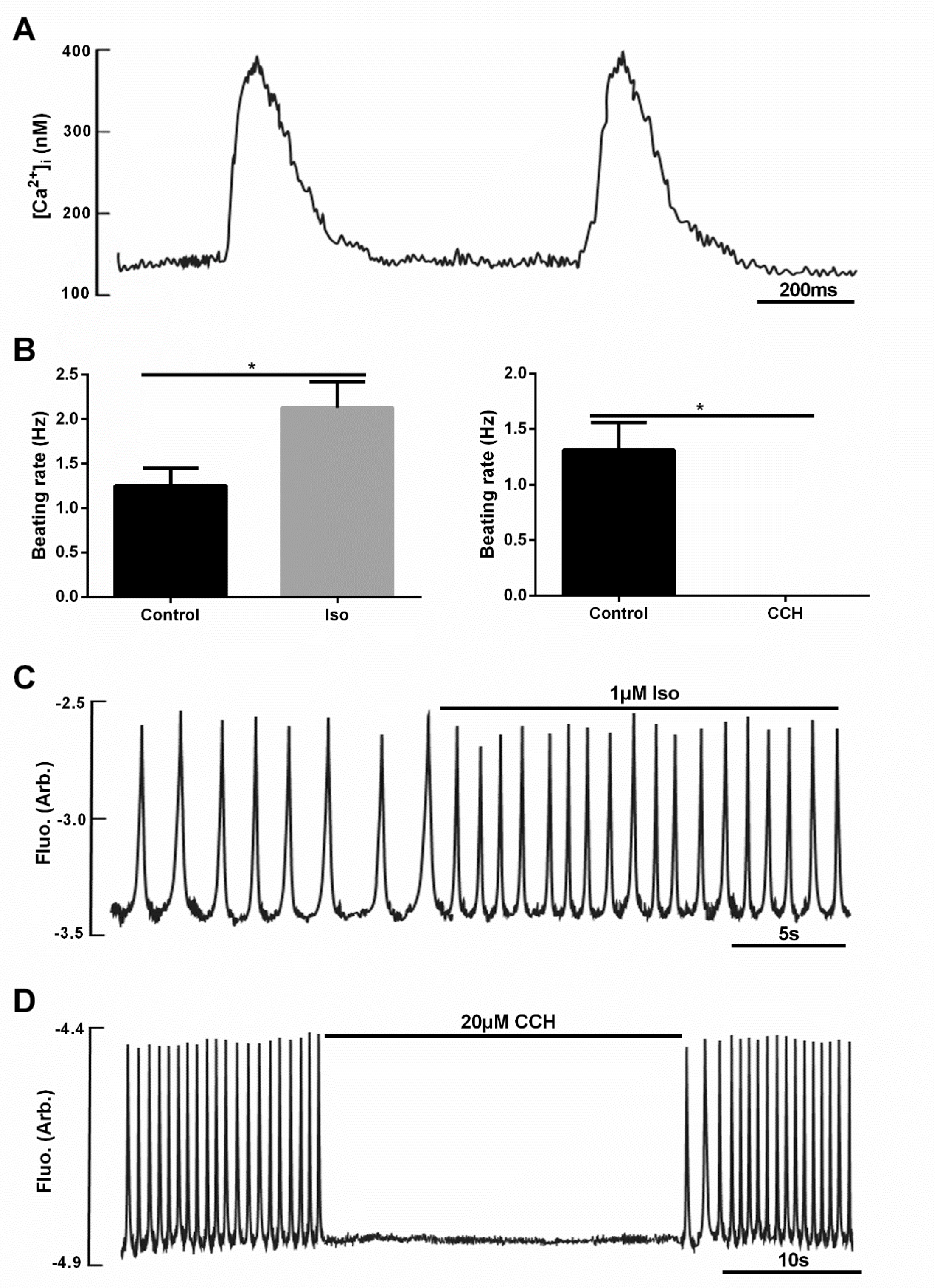

(A) Spontaneously contracting cardiomyocytes differentiated were recorded for the characteristic calcium transients ($[Ca^{2+}]_i$), and the representative curve was showed. (B) The effects of 1mM isoproterenol (Iso) (E and F) and 25mM carbachol (CCH) on the beating rates of enabled cardiomyocytes . (C, D) Calcium transients were compared before and after treatment 1mM Iso (C) and 25mM CCH) (G). Data are mean ± s.d. * $P<0.05$ compared with control.

Supplementary table 1 Primers used for qRT-PCR analysis

**KLF4**	**SOX1**
Forward: 5’- GATGAACTGACCAGGCACTA-3’;	Forward: 5’-ATACCCCCAAAATGCATCAA-3’;
Reverse: 5’- GTGGGTCATATCCACTGTCT-3’.	Reverse: 5’-GGAAACGGGCTTTTCTCTCT-3’.
**4-Oct**	**PAX6**
Forward: 5’-TCAGTGATGCTGTTGATCAGG-3’	Forward: 5’-GTTCCCTGTCCTGTGGACTC-3’;
Reverse: 5’-GCTATCTACTGTGTGTCCCAGTC-3’	Reverse: 5’-ACCGCCCTTGGTTAAAGTCT-3’.
**Nanog**	**Notch1**
Forward: 5’-AAACCAGTGGTTGAAGACTAGCAA-3’;	Forward: 5’-TGCCAGACCAACATCAAC-3’;
Reverse: 5’-GGTGCTGAGCCCTTCTGAATC-3’.	Reverse: 5’-CTCATAGTCCTCGGATTGC-3’.
**Sox2**	**GAPDH**
Forward: 5’- CCGTTTTCGTGGTCTTGTTT-3’.	Forward: 5’-GGTGGTCTCCTCTGACTTCAACA-3’;
Reverse: 5’- TCAACCTGCATGGACATTTT-3’.	Reverse: 5’-GTGGTCGTTGAGGGCAATG-3’.
**FOXA2**	**NKX2.5**
Forward: 5’- GGCCCAGTCACGAACAAAGC-3’;	Forward: 5’-CAAGTGCTCTCCTGCTTTCC-3’;
Reverse: 5’-CCCAAAGTCTCCACTCAGCCTC-3’.	Reverse: 5’-GGCTTTGTCCAGCTCCACT-3’.
**SOX17**	**GATA4**
Forward: 5’-AAGAAACCCTAAACACAAACAGCG-3’;	Forward: 5’-TCTCACTATGGGCACAGCAG-3’;
Reverse: 5’-TTTGTGGGAAGTGGGATCAAGAC-3’.	Reverse: 5’-GCGATGTCTGAGTGACAGGA-3’.
**AFP**	**cTnT**
Forward: 5’-CCCGAACTTTCCAAGCCATA-3’;	Forward: 5’-CAGAGCGGAAAAGTGGGAAGA-3’;
Reverse: 5’-TACATGGGCCACATCCAGG-3’.	Reverse: 5’-TCGTTGATCCTGTTTCGGAGA-3’.
**MESP1**	**α-MHC**
Forward: 5’-TGTACGCAGAAACAGCATCC-3’;	Forward: 5’-CGCATCAAGGAGCTCACC-3’;
Reverse: 5’-TTGTCCCCTCCACTCTTCAG-3’.	Reverse: 5’-CCTGCAGCCGCATTAAGT-3’.
**Brachyury**	**β-MHC**
Forward: 5’-GCTTCAAGGAGCTAACTAACGAG-3’;	Forward: 5’-TATCGATGACCTGGAGCTGA-3’;
Reverse: 5’- CCAGCAAGAAAGAGTACATGGC-3’.	Reverse: 5’-AGTATTGACCTTGTCTTCCTC-3’.
**Mixl1**	**SRF**
Forward: 5’-GGAGCTCGTCTTCCGACAGA-3’;	Forward: 5’-AGTGCAGGCCATTCAAGT-3’;
Reverse: 5’-TTGAGGATAAGGGCTGAAATGAC-3’.	Reverse: 5’-ACGGATGACGTCATGATGGTG-3’.
